# Robustness of calibration model for prediction of lignin content in different batches of snow pears based on NIR spectroscopy

**DOI:** 10.3389/fpls.2023.1128993

**Published:** 2023-02-27

**Authors:** Xin Wu, Guanglin Li, Xinglan Fu, Weixin Wu

**Affiliations:** ^1^ School of Electronics and Internet of Things, Chongqing College of Electronic Engineering, Chongqing, China; ^2^ College of Engineering and Technology, Southwest University, Chongqing, China; ^3^ Mechanical Measurement and Testing Research Center, Academy of Metrology and Quality Inspection, Chongqing, China

**Keywords:** lignin content of snow pears, robustness, SS-FPME method, NIR spectroscopy, calibration model

## Abstract

Snow pear is very popular in southwest China thanks to its fruit texture and potential medicinal value. Lignin content (LC) plays a direct and negative role (higher concentration and larger size of stone cells lead to thicker pulp and deterioration of the taste) in determining the fruit texture of snow pears as well as consumer purchasing decisions of fresh pears. In this study, we assessed the robustness of a calibration model for predicting LC in different batches of snow pears using a portable near-infrared (NIR) spectrometer, with the range of 1033–2300 nm. The average NIR spectra at nine different measurement positions of snow pear samples purchased at four different periods (batch A, B, C and D) were collected. We developed a standard normal variate transformation (SNV)-genetic algorithm (GA) -the partial least square regression (PLSR) model (master model A) - to predict LC in batch A of snow pear samples based on 80 selected effective wavelengths, with a higher correlation coefficient of prediction set (Rp) of 0.854 and a lower root mean square error of prediction set (RMSEP) of 0.624, which we used as the prediction model to detect LC in three other batches of snow pear samples. The performance of detecting the LC of batch B, C, and D samples by the master model A directly was poor, with lower Rp and higher RMSEP. The independent semi-supervision free parameter model enhancement (SS-FPME) method and the sequential SS-FPME method were used and compared to update master model A to predict the LC of snow pears. For the batch B samples, the predictive ability of the updated model (Ind-model AB) was improved, with an Rp of 0.837 and an RMSEP of 0.614. For the batch C samples, the performance of the Seq-model ABC was improved greatly, with an Rp of 0.952 and an RMSEP of 0.383. For the batch D samples, the performance of the Seq-model ABCD was also improved, with an Rp of 0.831 and an RMSEP of 0.309. Therefore, the updated model based on supervision and learning of new batch samples by the sequential SS-FPME method could improve the robustness and migration ability of the model used to detect the LC of snow pears and provide technical support for the development and practical application of portable detection device.

## 1 Introduction

Snow pear enjoys widespread popularity in southwest China (Wang et al., 2020; Wu et al., 2021). It has excellent fruit texture and boasts some medicinal value (Zou, 2016). Lignin content (LC), however, has a direct and negative effect on the fruit texture of snow pears and on consumers’ decision to purchase fresh pear fruit (Tao et al., 2009; Cai et al., 2010; Yan et al., 2014; Xue et al., 2019; Sheng et al., 2020; Wu et al., 2021). More specifically, higher concentration and larger size of stone cells lead to thicker pulp and deterioration of the taste. In recent decades, the use of near-infrared (NIR) spectroscopy has been an effective tool for the nondestructive and rapid detection of the internal quality of fruits and vegetables (Xiaobo et al., 2010). In particular, NIR spectroscopy, combined with the chemometric methods, has been successfully used to predict the soluble solids content (SSC), firmness, and moisture of fruits (e.g., apples, pears, tomatoes, peaches) by notable researchers (Zhang et al., 2008; Rahman et al., 2017; Tian et al., 2018; Du et al., 2019). Although the author and other researchers have studied the calibration model to predict the LC of snow pears based on NIR spectroscopy (Sheng et al., 2020; Wu et al., 2021), the robustness and accuracy of this model need further study and more research to assess for variability of samples and external variability of the measurement systems.

To obtain more stable and robust prediction results, researchers typically have used partial least square regression (PLSR) to establish calibration models based on the effective wavelengths from the full NIR spectra for predicting the internal quality of fruits and vegetables. The leave-one-out cross-validation method has been used to avoid overfitting or underfitting by using too many or too few PLS components in the PLSR algorithm, respectively (Douglas et al., 2018). The optimal number of latent variables (LVs) was determined by a full cross-validation of the calibration samples and an optimal number was determined by the minimum value of the root mean square error of cross-validation (RMSECV). The full-spectra PLSR model, however, was time-consuming, redundant, and collinear (Rahman et al., 2018). We used the variables selection method to extract the effective wavelengths and were able to reduce the complexity and increase the predictive ability of the PLSR model to detect the internal quality of fruits and vegetable (Xiaobo et al., 2010; Balabin and Smirnov, 2011; Xu et al., 2012; Jie et al., 2013; Deng et al., 2014; Li et al., 2014). In recent years, many effective wavelengths selection methods have been studied to predict internal quality based on NIR spectroscopy. Tao used the successive projection algorithm (SPA) to selected five optimal wavelengths for exploring an accurate and non-destructive method to discriminate the sex of silkworm pupae using the visible and near-infrared hyperspectral imaging technique (Tao et al., 2019). Li used the synergy interval partial least squares (SiPLS) combining with nonlinear SVM to developed a rapid quantitative analysis model for determining the glycated albumin content based on the attenuated total reflection–Fourier transform infrared (ATR-FTIR) spectroscopy (Li et al., 2018). Du used the genetic algorithm (GA) to optimize non-destructive prediction on property of mechanically injured peaches during postharvest storage by portable visible/shortwave near-infrared spectroscopy (Du et al., 2019). Deng developed the bootstrapping soft shrinkage (BOSS) method for variable selection in chemical modeling, and the method was used to select key variables for measurement moisture, oil, protein, and starch of corn and soy (Deng et al., 2016). Yan proposed a new computational method stabilized bootstrapping soft shrinkage approach (SBOSS) for variable selection based on the BOSS method for spectral variable selection in the issue of over-fitting, model accuracy and variable selection credibility (Yan et al., 2019). The competitive adaptive reweighted sampling (CARS) is an effective method for selecting effective wavelengths for multivariate calibration (Li et al., 2009; Jiang et al., 2015). Wang used the CARS to identify the characteristic wavelengths and simplify the PLS models for detection of juiciness of pear *via* VIS/NIR spectroscopy (Wang et al., 2020). Yang used the CARS to select feature variables for identification of unhealthy panax notoginseng from different geographical origins based on ATR-FTIR spectroscopy (Yang et al., 2019). Liang used the CARS to extract effective wavelengths for prediction of holocellulose and lignin content of pulp wood feedstock using NIR spectroscopy (Liang et al., 2020). The CARS has been also used to select variables for predicting internal quality of orange, dovyalis fruit, and pears by Song (Song et al., 2020), Mateus (de Assis et al., 2018), and Wu (Wu et al., 2021), respectively. In this work, these variables selection methods were used to extract effective wavelengths from the full NIR spectrum.

The prediction results of one master calibration model to measure the LC of different batches of snow pear samples has always had large errors based on NIR spectroscopy (Nicolaï et al., 2008). The “different batches” usually referred to the different measurement times, different seasons, different geographical locations, and different fruit maturity of snow pear samples (Anderson et al., 2021). Moreover, changes in the ambient temperature of NIR spectrum acquisition and the instrument components (such as the light source) could affect the accuracy and robustness of the calibration model. Therefore, the prediction ability of the model has to be checked routinely, because the NIR spectrum data was affected by the possible failures of the mechanical modules of the NIR spectrometer system (e.h., sensors, light sources, reference modules) in the process of collecting NIR spectra (Mercader and Puigdomenech, 2014). In addition, the error of calibration model measuring the corresponding LC of a new batch of snow pear samples has been significant for two reasons: (1) the NIR spectrum of this new batch missed the feature information corresponding to the measurement LC (Anderson et al., 2020); and (2) the external effect of the new batch of snow pear samples produced interference with NIR spectral information (Zeaiter et al., 2006). These variabilities in spectral information were related to the different varieties of samples, harvest season, and measured temperature. Therefore, to accurately predict the LC of a new batch of snow pears, in this work, we updated the calibration model using a semi supervision free parameter model enhancement (SS-FPME). The objective of this work was to analyze the accuracy and robustness of the calibration model to predict the LC of different batches of snow pears based on NIR spectroscopy. We proposed and applied the SS-FPME to update the PLSR model. The research processes of this work are as follows: (1) The NIR diffuse reflectance spectrum of four batches snow pear samples were obtained by an optic-spectrometer system. (2) We built a calibration model for the measurement of the LC of snow pears based on the most effective wavelengths from the full spectrum of the optimal measurement positions of samples selected by the SPA, SiPLS, GA, BOSS and CARS methods. (3) The SS-FPME method was used to update the calibration model to predict the LC of batch B, C, and D, and we compared and analyzed two ways to update the model. (4) We evaluated the performance of the PLS model based on the independent verification data sets.

## 2 Materials and methods

### 2.1 Samples preparation

A total of 512 snow pears of four different batches of samples were collected from the local fruit market at different time periods in Shuangfu, Chongqing. The surface of these samples did not bear any damage. The average fruit weight was 300–400 g. The shape was round or flat, with the top and base uneven, the longitudinal diameter around 8–9 cm, the transverse diameter around 9–9.5 cm, and the fruit stone diameter of 2–3.5 cm. After each batch of samples was collected and brought back to the laboratory, the snow pears were washed, numbered, and stored in a refrigerator to ensure the accuracy of the experiment. It took eight months to collect the NIR diffuse reflectance spectra of the surface of the samples using a microfiber spectrometer and to measure the standard reference values of the LC according to the Klason method (Bunzel et al., 2011; Cybulska et al., 2012; Assis et al., 2017). Among these samples, the NIR spectra and LC reference value of the 160 samples in batch A were completed in December 2020, and the 120 samples in batch B, 104 samples in batch C, and 128 samples in batch D were completed in March 2021, May 2021, and July 2021, respectively. Different batches of samples in this research referred to the different collection time points of NIR diffuse reflection spectrum of the samples. As shown in **Table 1**, the batch A samples were divided into a calibration set (60%) and a validation set (40%) using the Kennard–Stone (KS) algorithm (Tao et al., 2019), and the batch B, C, and D samples were divided into a model update calibration set (40%) and a validation set (60%).

**Table 1:** Statistical data of lignin content (mg/g) of snow pear samples of four batches.

Batch	Measurement time	Data set	Number	Range (mg/g)	Mean ± SD (mg/g)	SEL
A	2020.12	All samples	160	75.05–81.04	77.87 ± 1.22	0.096
		Calibration set	96	75.05–81.02	77.59 ± 1.20	0.109
		Prediction set	64	75.85–81.04	78.27 ± 1.14	0.143
B	2021.03	All samples	120	74.78–80.80	77.75 ± 1.18	0.108
		Calibration set	48	74.78–79.99	77.14 ± 1.09	0.157
		Prediction set	72	76.27–80.80	78.16 ± 1.07	0.126
C	2021.05	All samples	104	75.48–81.42	78.03 ± 1.19	0.116
		Calibration set	42	75.68–80.25	77.51 ± 1.10	0.170
		Prediction set	62	75.48–81.42	78.39 ± 1.12	0.142
D	2021.07	All samples	128	76.43–79.38	77.93 ± 0.55	0.048
		Calibration set	51	76.76–78.67	77.73 ± 0.49	0.068
		Prediction set	77	76.43–79.38	78.06 ± 0.54	0.062

SD, standard derivation; SEL, standard error of laboratory.

### 2.2 Spectral measurement

**Figure 1:**
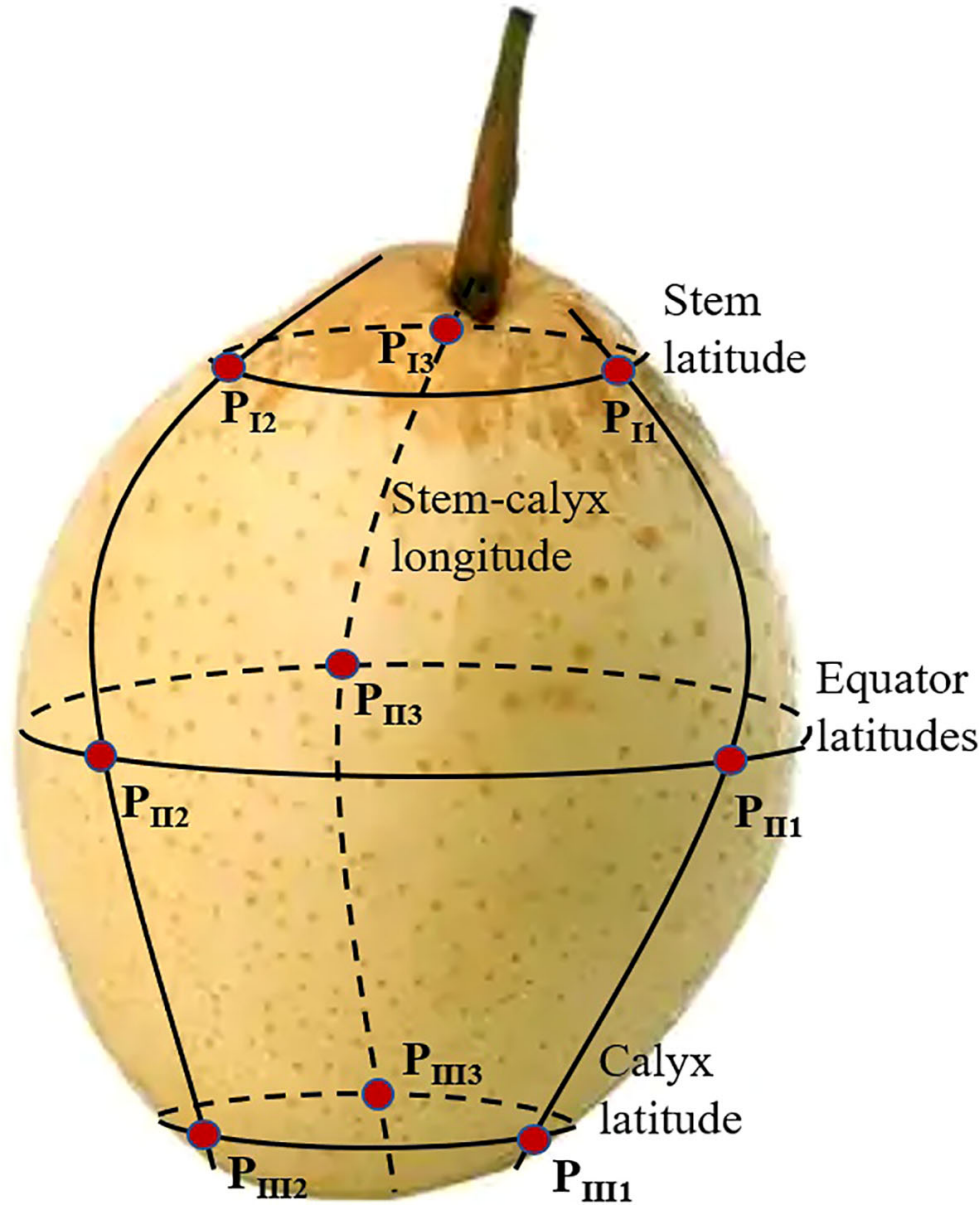
Diagram of the nine spectral measurement positions of one sample. The first longitude intersects the stem latitudes, equator latitudes, and calyx latitudes form three spectral measurement positions: P_I1_, P_II1_, and P_III1_. The second longitude and third longitude intersect to form six spectral measurement positions: P_I2_, P_II2_, and P_III2_, and P_I3_, P_II;3_, and P_III3_, respectively.

Based on the NIR diffuse reflectance spectrum acquisition system, the NIR spectra of nine measurement positions (three stem-calyx longitude, with an interval of 120°) intersected three latitudes (stem, equator, and calyx) from nine spectral measurement positions (as shown in **Figure 1**) on the surface of four batches of snow pears that were collected using a microfiber spectrometer (NIRQuest256-2.5, Ocean Insight, Orlando, FL, USA). The microfiber optic spectrometer had wavelengths ranging from 900 to 2500 nm, with a resolution of 9.5 nm and 512 data points. We set the integration time of the microfiber optic spectrometer to 70 ms, the scanning number to 5, and the number of smoothing points to 10. We obtained the average NIR spectrum of one sample after three consecutive acquisitions at each measurement point. The noise spectral data at both ends of the spectral curve were removed, and the effective wavelengths ranged from 1033 to 2300 nm, with 387 spectral points.

### 2.3 Reference LC measurement

To make the spectrum and LC correspond more accurately, the fresh snow pear flesh (between 2 cm outside the core and 2 mm under the pericarp of an intact pear) was made into a dry powder immediately after the NIR spectrum acquisition. We used the traditional Klason method to measure the LC reference value of snow pears, and the statistical results are shown in **Table 1**. The snow pear dry powder (500 mg) and 72% H_2_SO_4_ (30 mL) formed the mixed solution; the solution was stirred evenly, sampled in boiling water bath for 2 h, and diluted with deionized water. Then, the solution was poured into a sand core funnel (diameter of 2.5 cm, particle retention of 1.6 μm), filtrated, washed, dried, and weighed to obtained the LC mass ratio (mg/g) of the sample. We conducted three chemical repeated measurements and obtained the value with a relative error within 5% was obtained.

The LC values of snow pear samples of batches A, B, C, and D ranged from 75.05 to 81.04 mg/g, 74.78 to 80.80 mg/g, 75.48 to 81.42 mg/g, and 76.43 to 79.38 mg/g, respectively. **Table 1** also shows the lignin distribution of the calibration set and the prediction set, and the LC range in the calibration set was bigger than that in the prediction set for the batch A samples. This result was helpful to build a better calibration model for detecting the LC of snow pears in batch A.

### 2.4 Theory of SS-FPME

For the multivariate calibration model, it was assumed that a data set of NIR spectrum was **X**_(mxn)_, the number of samples was m, the number of variSSables was n, and the data set of the LC reference value was **Y**_(mx1)_. The linear relationship between **X** and y can be established by the PLSR model, as shown in formula (1). The predicted value $\hat{y}$ could be calculated, as follows:


(1) $$y=\left[1X\right]\left[\begin{array}{c} b_{0}\\ b \end{array}\right]+e=\hat{y}+e$$

where *b*_0_ and b(*n*x1) were the intercept and regression coefficient of the PLS model, respectively; 1 was the column vector of length n, and its element was 1; and **e** was the prediction error between **ŷ** and **y**.

If only data sets for the NIR spectra and the LC reference value of the new batch of snow pear samples were available, and no data set was available for the NIR spectral of samples of the main batch, it would be impossible to update the calibration model to predict the LC of a new batch of snow pears using the standard strategy. In practical applications, an updated calibration model is often necessary to predict the LC of new samples. Therefore, it was necessary to apply the semi-supervision free parameter model enhancement (SS-FPME) to the updated calibration model. This method reduced the influence of sample variability and external variability of measurement systems to obtain an accurate and robust prediction result. The function formula of SS-FPME was calculated as follows:


(2) $$\begin{array}{l} \begin{array}{c} \min\\ b_{0,s},b_{s} \end{array}\left(\|y-[1X_{s}]{\left[\begin{array}{c} b_{0,s}\\ b_{s} \end{array}\right]\|}^{2}\right)\\ s.t.corr(b_{s},b_{m})>r_{th} \end{array}$$

where X_*s*_ is the data set of the NIR spectra of samples of new batch and the updated data set of the calibration model at the same time; *b*_*0,s*_ is the intercept; b_s_ is the regression coefficient of calibration model of the new batch sample, and *r_th_* is the constraint of the correlation coefficient; and b*_m_* is the regression coefficient of calibration model of the original main batch sample, which could be analyzed and calculated by PLSR model. We optimized the function formula (2) of SS-FPME using the sequential quadratic programming method of the fmincon optimization routine of MATLAB 2016b software. The method to update the SS-FPME model required the regression coefficient of the primacy model, the spectral data set of a few samples from the new batch, and the data set of the corresponding reference value. We used the root mean square error of the prediction set (RMSEP) to evaluate the performance of the updated calibration model, which was estimated based on the independent test set.

### 2.5 Method of updating model method by SS-FPME

**Figure 2:**
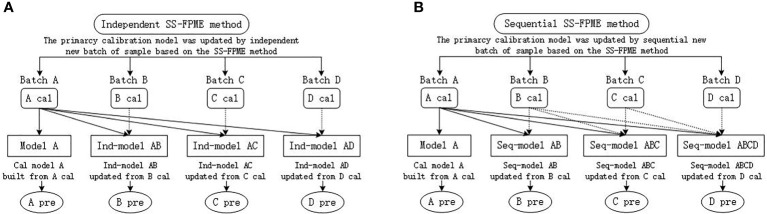
Schematic of calibration model updating method based on SS-FPME: **(A)** independent SS-FPME method and **(B)** sequential SS-FPME method. Cal, calibration; Pre, prediction.

To comprehensively assess the prediction ability of the updated calibration model of different batches of snow pears based on NIR spectroscopy, we used the SS-FPME method to update the calibration model of the old batch of samples based on the updated data set of the new batch of samples to predict the LC of the new batch of samples. We updated the master calibration model according to each new batch of samples independently in the SS-FPME method, referred to as the independent SS-FPME method (**Figure 2A**), and the master calibration model was updated sequentially by multiple batches of the samples, referred to as the sequential SS-FPME method (**Figure 2B**).

For the independent SS-FPME method, **Figure 2A**) shows the updating process for the calibration model to predict the LC in the four batches of snow pears. We used the PLSR to establish the master calibration model based on one batch of snow pear samples (batch A), and formed model A to predict the LC of batch A. To improve the accuracy of the calibration model, we had to update the master model (model A) from the calibration set of a new batch of samples (batch B), and formed Ind-model AB to predict the LC of batch B. The calibration set of the new batch of samples contained few samples, and was called the update set. To accurately detect the LC of batch C and batch D, we built the Ind-model AC and Ind-model AD from the calibration set of batch C and batch D independently using the SS-FPME method.

**Figure 2B** shows the updating process for the calibration model of the sequential SS-FPME method for four batches of snow pears. Similar to the independent SS-FPME method, we built model A (the master model) from the calibration set of batch A using the PLSR algorithm to predict the LC of batch A, and we updated model A to form the Seq-model AB from the calibration set of batch B to predict the LC of batch B. Then, we updated the Seq-model AB to form the Seq-model ABC from the calibration set of batch C sequentially to predict the LC of batch C, and we updated the Seq-model ABC to form the Seq-model ABCD from the calibration set of batch D sequentially to predict the LC of batch D. In this work, the independent SS-FPME method and the sequential SS-FPME method were used and compared to update the calibration model to predict the LC of four batches of snow pears separately to improve the accuracy and robustness of the calibration model to predict the internal qualities of different batches of samples.

### 2.6 Evaluation model

#### 2.6.1 PLSR modeling

The PLSR algorithm is a multivariate linear analysis method first proposed by Wold and Krishnaiah, which is widely used in the analysis of spectral data (Haaland and Thomas, 1988). The basic principle of this algorithm is to obtain the score matrix by decomposing the sample spectral matrix and sample concentration matrix at the same time and to perform multiple linear regression. Following are the main implementation steps of the PLSR. First, the principal components of spectral matrix X and concentration matrix Y of the sample are decomposed, as follows:


(3) $$X_{m\times n}=T_{m\times w}P_{w\times n}+E_{m\times n}$$

(4) $$Y_{m\times l}=U_{m\times w}Q_{w\times l}+F_{m\times l}$$

Where *X_mxn_* is the spectral matrix of *m* samples at *n* wavelengths; *Y_mx1_* is the concentration matrix containing the content information of *l* components of *m* samples; *T_mxw_* and *U_mxw_* are the score matrix; *P_wxn_* and *Q_mx1_* are the load matrix; and *E_wxn_* and *F_mx1_* are the residual matrix.

Then the linear regression of *T_mxw_* and *U_mxw_* are processed as follows:


(5) $$U_{m\times w}=T_{m\times w}\cdot B_{w\times w}$$

Where *B_wxw_* is the regression coefficient matrix:


(6) $$B_{w\times w}=U_{m\times w}\cdot T_{m\times w}^{T}{\left(T_{m\times w}T_{m\times w}^{T}\right)}^{-1}$$

#### 2.6.2 Model evaluation indexes

Generally, correlation coefficient and root mean square error are used as the evaluation indexes for NIR spectral data analysis, including the correlation coefficient of calibration set (Rc), the root mean square error of cross-validation (RMSECV), the correlation coefficient of prediction set (Rp), and the root mean square error of prediction set (RMSEP):


(7) $$R_{c}=\sqrt{1-\frac{\sum_{i=1}^{n}{\left(y_{i,a}-y_{i,p}\right)}^{2}}{\sum_{i=1}^{n}{\left(y_{i,a}-y_{i,cm}\right)}^{2}}}$$

(8) $$RMSECV=\sqrt{\frac{\sum_{i=1}^{n}{\left(y_{i,a}-y_{i,p}\right)}^{2}}{n}}$$

In the calibration set, *n* is the number of samples, *Y_i,a_* is the standard reference of the *i*-th sample, *Y_i,p_* is the predicted value of the *i*-th sample, and *Y_i,m_* is the average value of the standard reference of all samples:


(9) $$R_{p}=\sqrt{1-\frac{\sum_{j=1}^{m}{\left(y_{j,a}-y_{j,p}\right)}^{2}}{\sum_{j=1}^{m}{\left(y_{j,a}-y_{j,pm}\right)}^{2}}}$$

(10) $$RMSEP=\sqrt{\frac{\sum_{j=1}^{m}{\left(y_{j,a}-y_{j,p}\right)}^{2}}{m}}$$

In the prediction set, *m* is the number of samples, *Y_j,a_*is the standard reference of the *j*-th sample, *Y_j,p_* is the predicted value of the *j*-th sample, and *Y_j,pm_* is the average value of the standard reference of all samples. The prediction model has a better accuracy and robustness with the higher Rc and Rp (closer to 1), and smaller and closer the values of REMSCV and RMSEP.

## 3 Results and discussion

### 3.1 Master calibration model to predict LC

**Figure 3:**
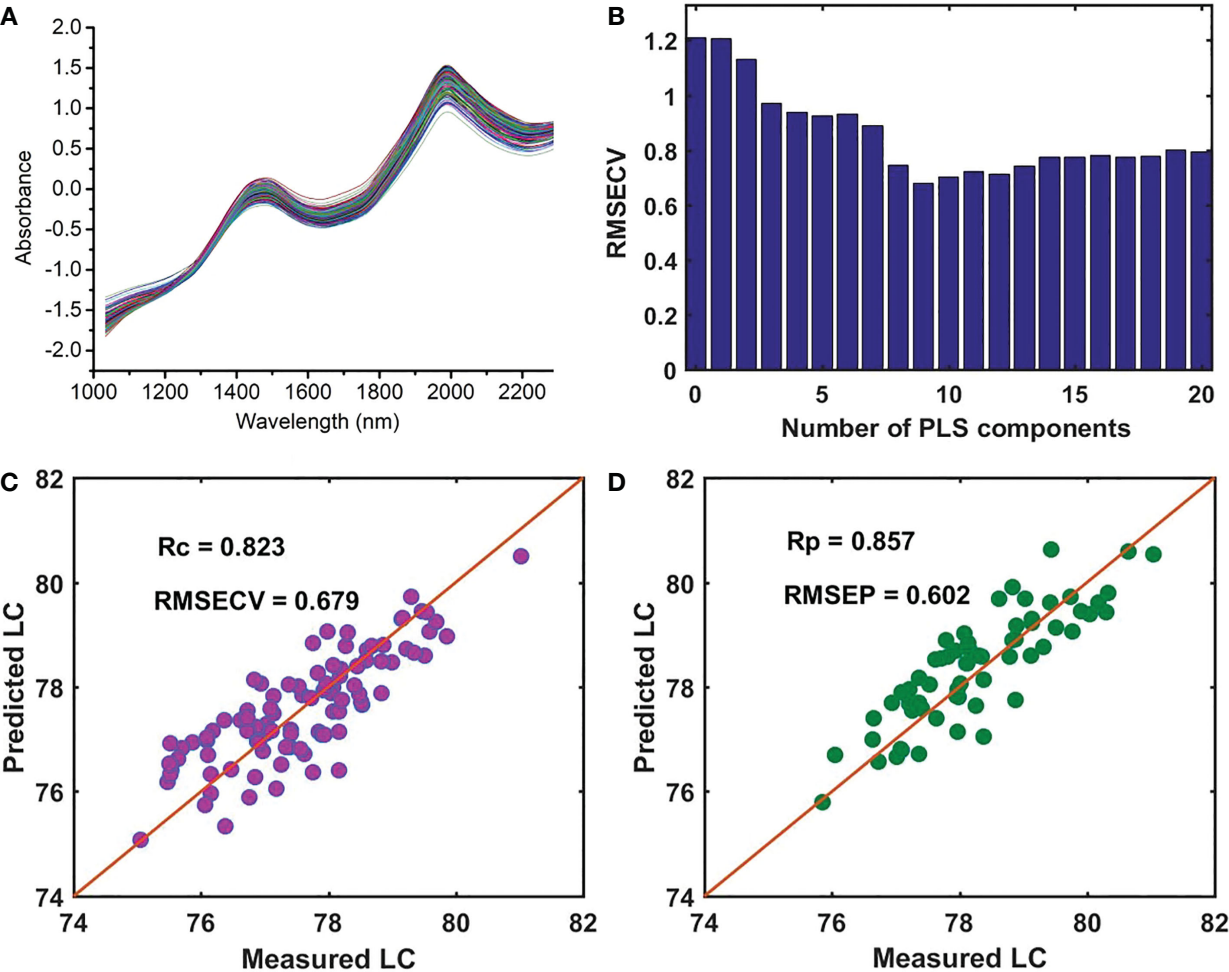
**(A)** Average spectra of each sample after SNV preprocessing, **(B)** the RMSECV versus the number of PLS components, **(C)** the performance of the PLSR model for measuring LC in the calibration set, and **(D)** the prediction set.

Based on NIR spectroscopy, we established the prediction model of the LC of snow pear samples in batch A, which was used as the master model for the detection of LC in four batches of samples in this study. To deduct the influence of instrument background or drift on the signal, eliminate random noise, and improve the signal-to-noise ratio, the first derivative (1-Der, polynomial order = 1, smoothing points = 11), second derivative (2-Der, polynomial order = 2, smoothing points = 11), standard normal variate transformation (SNV), and multiplicative scatter correction (MSC) were used and compared to pretreat the raw average NIR spectra of nine measurement positions at each sample. We carried out the preprocessing methods using the software Unscrambler X 10.4 (CAMO PRECESS AS, Oslo, Norway). The results shown in **Table 2** indicated that the prediction model using the SNV preprocessing method achieved better performance. Compared with the no preprocessing method, **Figure 3** showed that the Rc and Rp were improved from 0.807 and 0.850 to 0.822 and 0.857, respectively, whereas the RMSECV and RMSEP were reduced from 0.710 and 0.603 to 0.679 and 0.602, respectively. Therefore, we further analyzed the LC detection model based on the NIR data after SNV preprocessing.

**Table 2:** Performance of model based on the different preprocessing methods for measuring LC of batch A of samples.

Preprocessing method	Number of wavelengths	LVs	Rc	RMSECV	Rp	RMSEP
NONE	387	10	0.807	0.710	0.850	0.603
1-Der (11)	387	9	0.812	0.699	0.847	0.624
2-Der (11)	387	8	0.747	0.805	0.787	0.716
SNV	387	9	0.822	0.679	0.857	0.602
MSC	387	10	0.821	0.683	0.848	0.618

**Figure 4:**
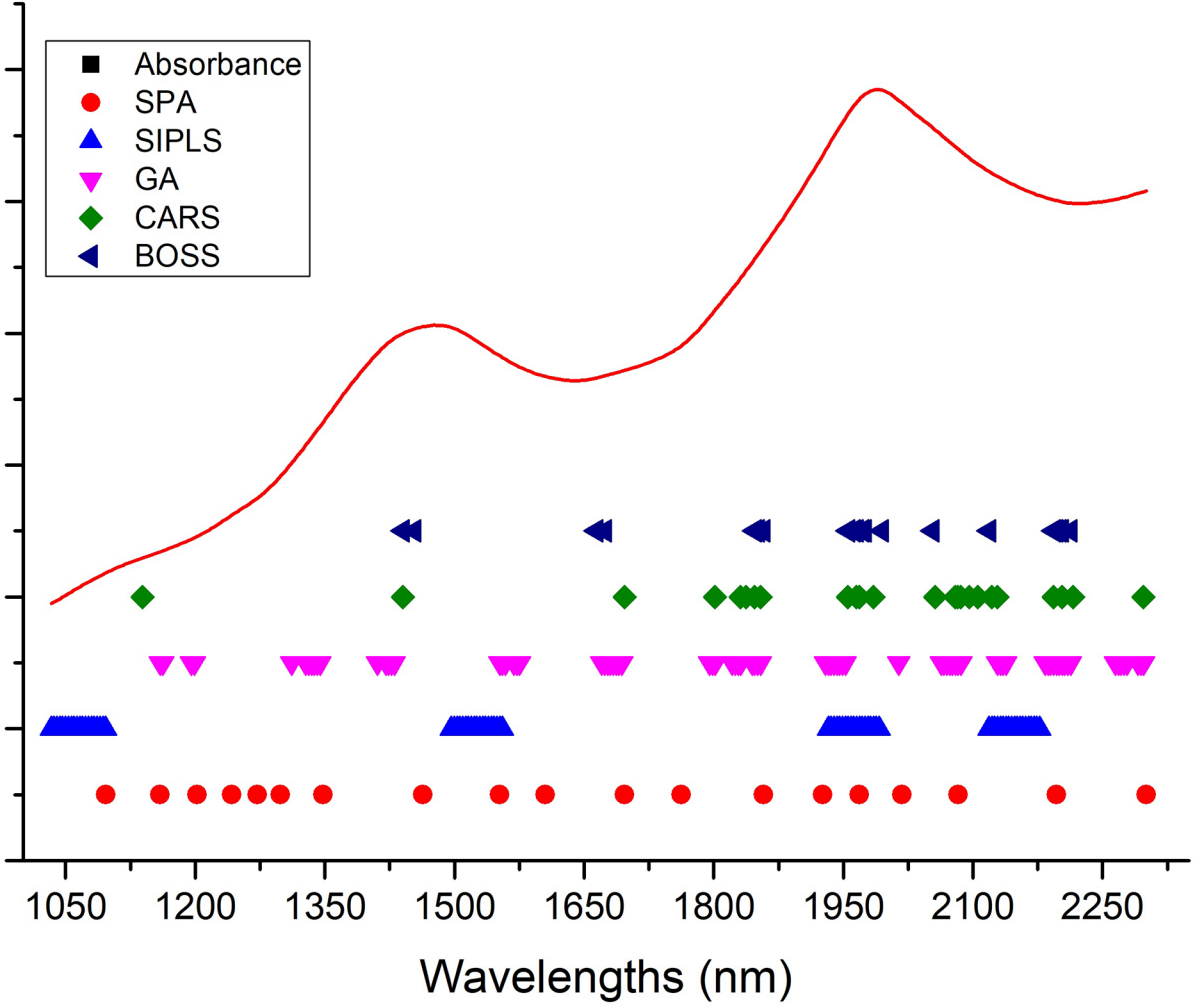
Distribution of effective wavelengths selected by SPA, SiPLS, GA, BOSS, and CARS methods.

Hundreds or thousands of wavelengths in the full spectra of samples may contribute more collinearity and redundancies and contain useless or irrelevant information. This makes the calibration process more time-consuming, is less convenient to meet high-speed spectroscopy features, and reduces the prediction accuracy of the calibration model to measure the LC of snow pears. To eliminate the uninformative wavelengths, predigest the calibration model, and improve the prediction results in terms of accuracy and robustness, we selected and compared 19, 76, 80, 24, and 20 effective wavelengths (as shown in **Figure 4**) to build a model to predict the LC of snow pears using the successive projections algorithm (SPA), synergy interval partial least squares (SiPLS), genetic algorithm (GA), bootstrapping soft shrinkage (BOSS), and competitive adaptive reweighted sampling (CARS) methods, respectively.

In the SiPLS method, we divided the full spectra into 20 subintervals, and selected the 1st, 8th, 15th, and 18th subintervals as the effective regions. During the process of CARS effective wavelengths selection, we set the number of Monte Carlo sampling runs, the maximal principal value, and the number of cross validation to 100, 10, and 10, respectively. The number of iterations and cross-validation of the BOSS algorithm were set to 2000 and 5, and the maximum number of latent variables was set to 20. The statistical data in **Table 3** show that the number of latent variables (LVs) of the model (SNV-CARS-PLSR) established based on the effective wavelengths selected by the CARS method was the lowest, which was eight LVs. The Rc of model (SNV-GA-PLSR) obtained by the GA method was the highest, which was 0.846, the Rp of the model (SNV-SPA-PLSR) by the SPA method was the highest (0.863), and the RMSECV and RMSEP values of the model (SNV-GA-PLSR) by the GA method were the lowest (0.637 and 0.624).

**Table 3:** Performance of the model based on different variables selection methods to measure the LC of batch A samples.

Variables selection method	Number of effective wavelengths	LVs	Rc	RMSECV	Rp	RMSEP
NONE	387	9	0.822	0.679	0.857	0.602
SPA	19	9	0.828	0.670	0.863	0.639
SIPLS	76	11	0.816	0.692	0.782	0.898
GA	80	10	0.846	0.637	0.854	0.624
CARS	24	8	0.840	0.647	0.859	0.645
BOSS	20	12	0.809	0.704	0.806	0.691

**Figure 5:**
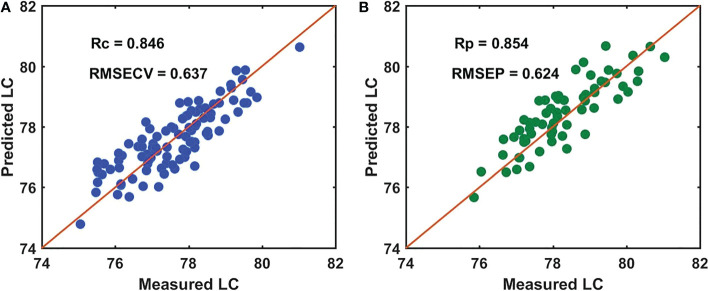
Performance of the SNV-GA-PLSR model for measuring the LC of batch A samples. **(A)** the calibration set, and **(B)** the prediction set.

According to the results, the SNV-GA-PLSR model (master model A) had higher Rc and Rp values of 0.846 and 0.854 and lower RMSECV and RMSEP values of 0.637 and 0.624 (as shown in **Figure 5**), respectively. Moreover, the difference between the Rc and Rp and the RMSECV and RMSEP also was smaller. Therefore, the SNV-GA-PLSR demonstrated better prediction performance for measuring the LC of snow pears, which we used as the prediction model for the four batches of snow pear samples in this study.

**Figure 6:**
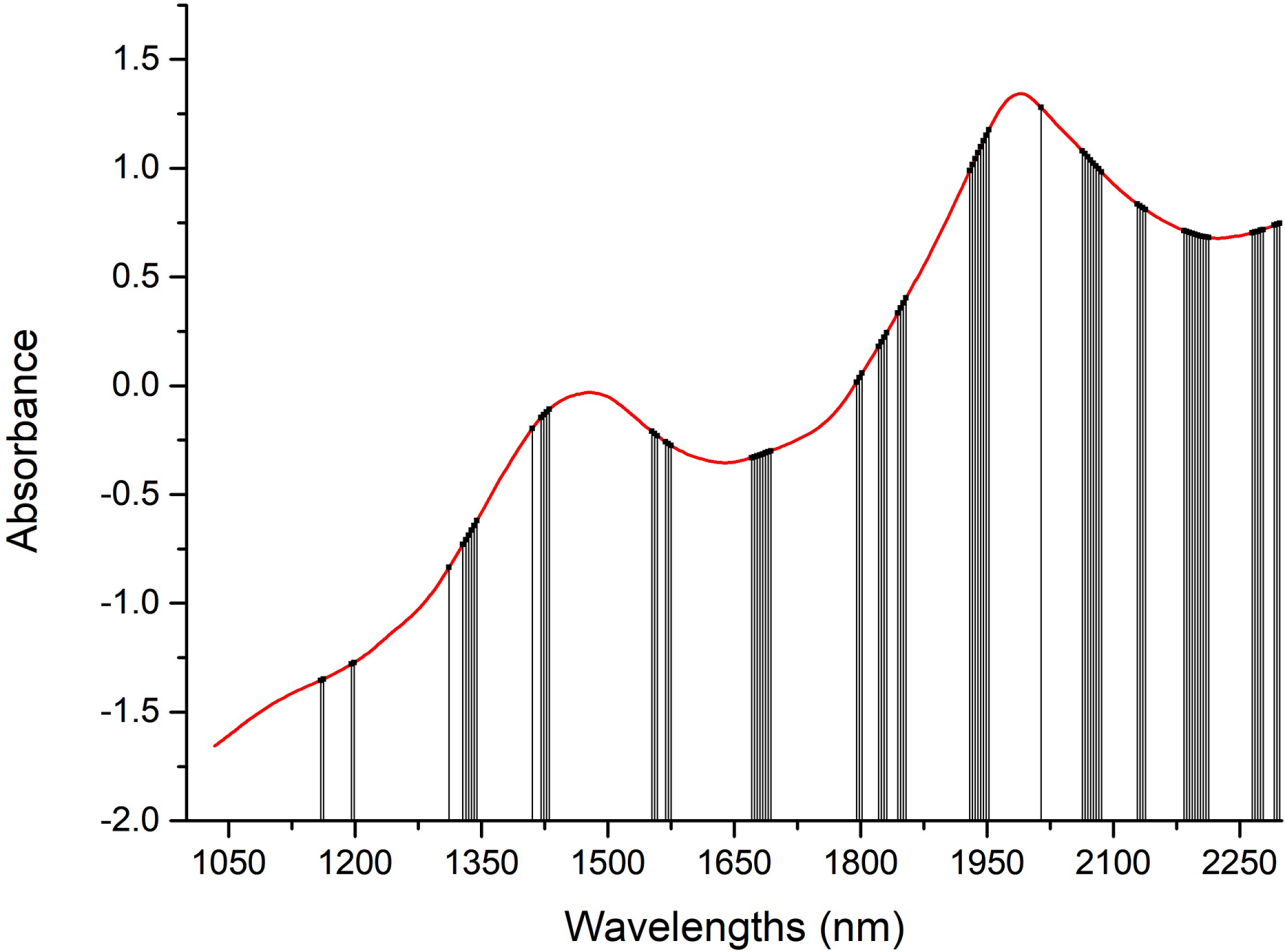
Distribution of the 80 effective wavelengths selected by the GA method.

As shown in **Figure 6**, the 80 selected effective wavelengths were distributed mainly at 1160 nm, 1198 nm, 1328–1344 nm, 1420–1430 nm, 1552–1575 nm, 1670–1693 nm, 1798 nm, 1821–1831 nm, 1844–1854 nm, 1929–1952 nm, 2063–2086 nm, 2128–2138 nm, 2183–2212 nm, 2264–2277 nm, and 2290–2300 nm. The NIR spectral region primarily contained the frequency doubling and combination bond information for C-H, N-H, and O-H, which was sensitive to the concentrations of organic materials. LC is the organic molecule and the C-H, N-H, and O-H were the most important groups with the main active ingredients. Thus, it is possible to use NIR methods for determination of LC in snow pear. Of these, 1160 nm and 1198 nm were associated with the third overtone of C-H; 1420–1430 nm was associated with the second overtone of the H2O, O-H, N-H, and C-H combination; 1552–1575 nm was associated with the first overtone of N-H; 1670–1693 nm and 1798 nm were associated with the first overtone of C-H; 1821–1831 nm was associated with the second overtone of the C=O stretch; 2063–2086 nm was associated with the H2O and O-H combinations; 2128–2138 nm was associated with the N-H combinations; 2183–2212 nm was associated with the N-H+C-C combinations; and 2264–2277 nm and 2290–2300 nm were associated with the C-H+C-H combinations.

The **Table 4** showed that SNV-GA-PLSR model can also simply the calibration model and improve the prediction performance for measuring the lignin content of batch B, C and D snow pears.

**Table 4:** Performance of the model based on GA method to measure the LC of batch B, C and D samples.

Batch	Variables selection method	Number of effective wavelengths	LVs	Rc	RMSECV	Rp	RMSEP
B	NONE	387	9	0.760	0.757	0.780	0.717
	GA	80	10	0.798	0.698	0.807	0.674
C	NONE	387	9	0.846	0.600	0.857	0.694
	GA	80	10	0.867	0.550	0.937	0.411
D	NONE	387	9	0.693	0.039	0.657	0.412
	GA	80	10	0.717	0.373	0.756	0.353

### 3.2 Robustness of the updated model by SS-FPME method

**Figure 7:**
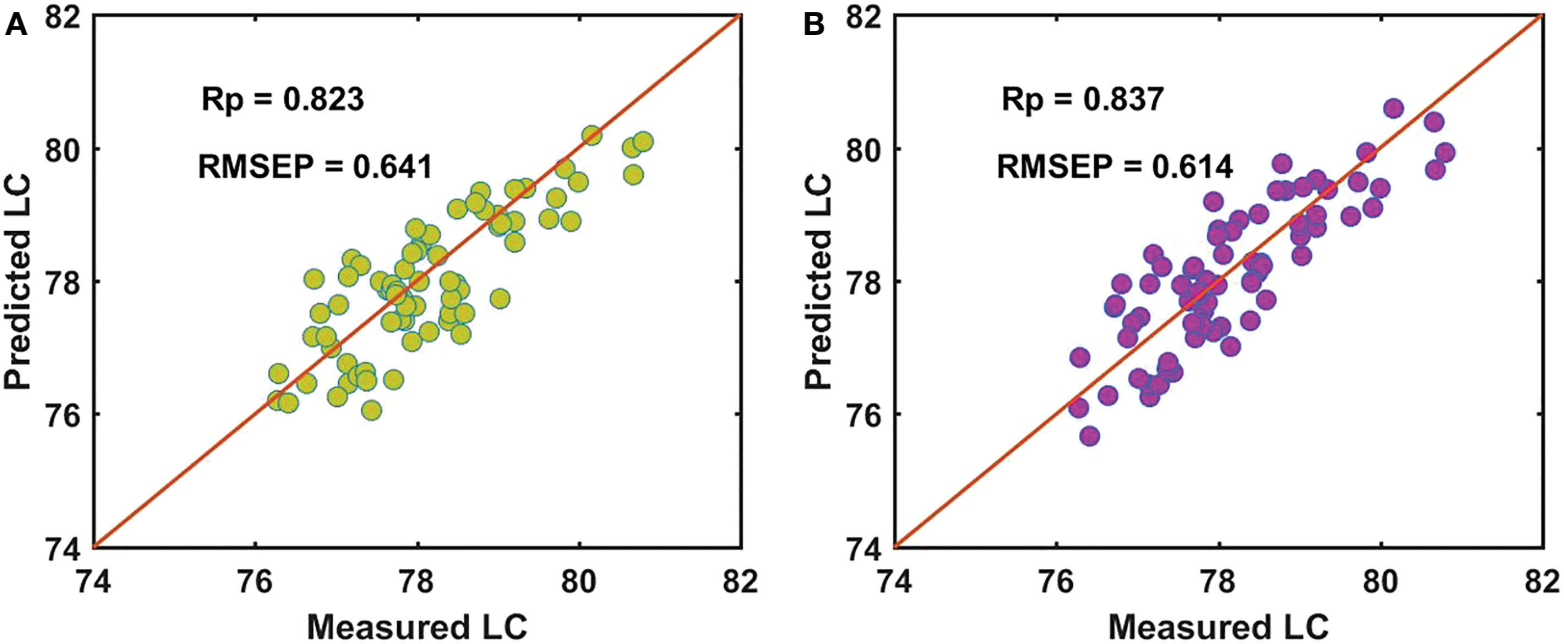
Performance for predicting LC of batch B of samples by **(A)** master model A and **(B)** Ind-model AB.

For the batch B samples of snow pears, we used master model A to directly measure the LC of the prediction data set of the batch B samples (Bpre), with the Rp of 0.823 and RMSEP of 0.641, as shown in **Figure 7A**. Based on the independent SS-FPME method, we obtained a new regression coefficient matrix (bs_AB) by using the regression coefficient matrix of master model A (bm_A) to supervise the learning of the calibration data set of the batch B samples (Bcal). Ind-model AB was established to predict the LC of Bpre, and the predictive ability of the updated model (Ind-model AB) was improved to a certain extent. **Figure 7B** shows that the Rp value increased from 0.823 to 0.837, and the RMSEP value decreased from 0.641 to 0.614.

**Figure 8:**
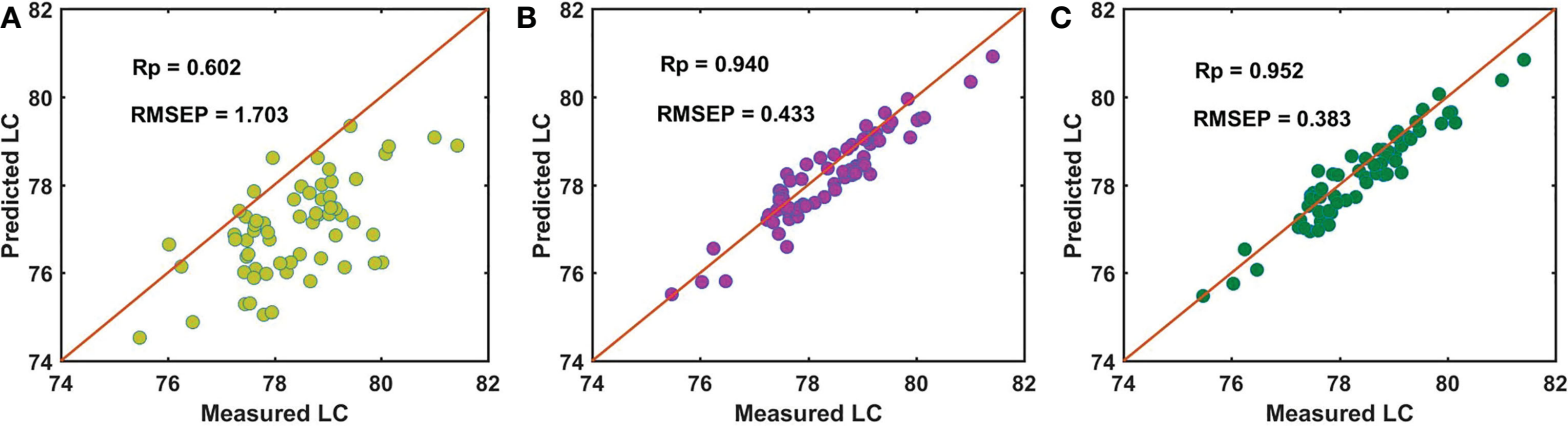
Performance for predicting LC of batch C of samples by **(A)** master model A, **(B)** Ind-model AC, and **(C)** Seq-model ABC.

For the batch C samples of snow pears, **Figure 8A** shows that the performance of using master model A to directly detect the LC of the prediction data set of the batch C samples (Cpre) was poor, with an Rp of 0.602 and RMSEP of 1.703. Based on the independent SS-FPME method, we obtained the regression coefficient matrix (bs_AC) and the Ind-model AC using the bm_A constraint supervision to learn the calibration data set of the batch C samples (Ccal). The prediction performance was greatly improved, with an Rp of 0.940 and RMSEP of 0.433, as shown in **Figure 8B**. Based on the sequential SS-FPME method, we used the regression coefficient matrix (bm_A) of master model A in supervised learning Bcal to first construct the bs_AB, and then we used the bs_AB in supervised learning Ccal to construct bs_ABC, and established the Seq-model ABC to measure the LC of the prediction data set of the batch C samples (Cpre). Compared with the Ind-model AC, the prediction performance was further improved: the Rp value increased from 0.940 to 0.952 and the RMSEP value decreased from 0.433 to 0.383, as shown in **Figure 8C**.

**Figure 9:**
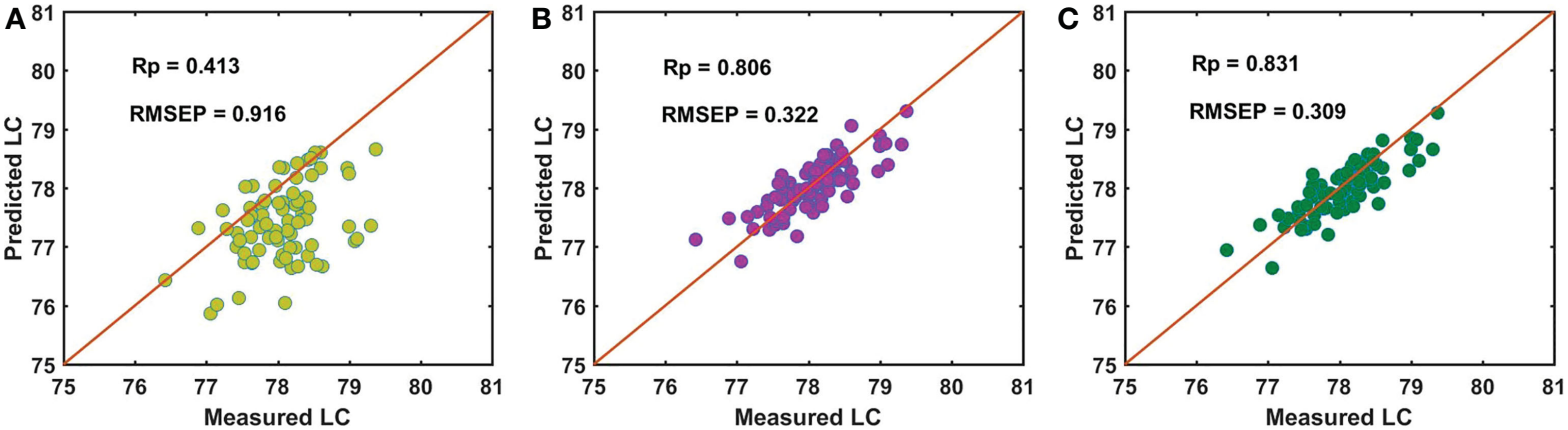
Performance for predicting LC of batch D of samples by **(A)** master model A, **(B)** Ind-model AD, and **(C)** Seq-model ABCD.

The analysis process for the batch D samples was the same as that for the batch C samples, and the experimental results are shown in **Figure 9**. First, master model A was directly used to measure the LC of the batch D samples, and the performance was poor, with the Rp of 0.413 and RMSEP of 0.916 (**Figure 9A**). Then, we built the Ind-model AD based on the calibration data set of the batch D samples (Dcal) and bm_A in the independent SS-FPME method. The Rp and RMSEP of the Ind-model AD to detect the LC of the prediction data set of the batch D samples (Dpre) were 0.806 and 0.322 (**Figure 9B**), respectively. For the sequential SS-FPME method, we built the bs_ABCD and Seq-model ABCD by updating the Seq-model ABC based on the Dcal and bs_ABC. The Rp and RMSEP of Seq-model ABCD were 0.831 and 0.309 (**Figure 9C**), respectively. Therefore, the sequential SS-FPME method updated the master model based on SS-FPME supervised learning of the new batch samples further increased the Rp and reduced the RMSEP of prediction model to measure the LC of the batch C and D samples, and further improved the prediction performance of the updated calibration model. Moreover, the prediction performance of the updated model based on the sequential SS-FPME method was better than that of the independent SS-FPME method. This result indicated that sequential update enhanced the model features in the learning of previous batches.

**Figure 10:**
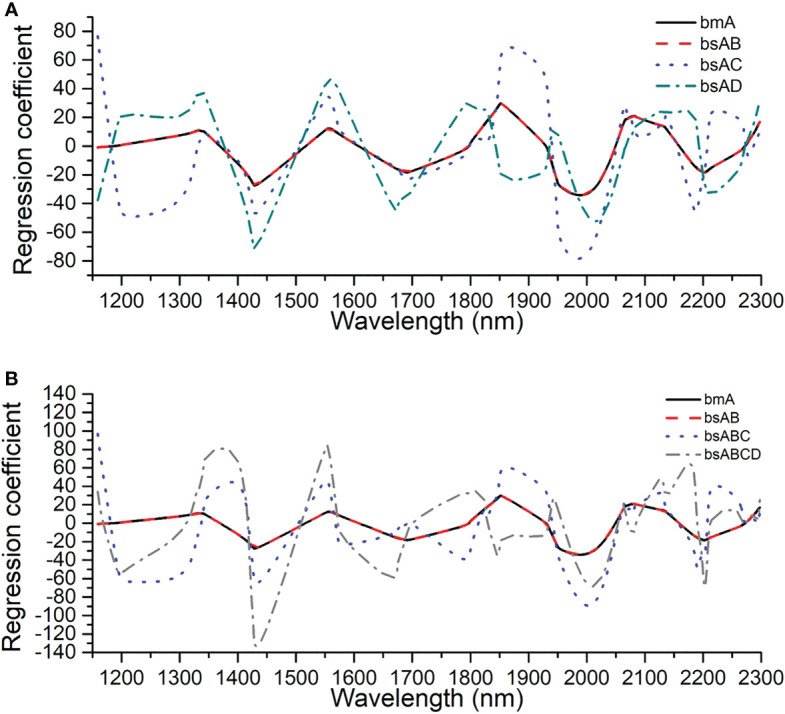
Evolution process of regression coefficients of master model A in **(A)** the independent SS-FPME method and **(B)** the sequential SS-FPME method.

The constraint condition of regression coefficient had to be adjusted in the process of updating the master model using the independent SS-FPME method and the sequential SS-FPME method, which contained the information variation of the NIR spectra in the current batch and the new batch of snow pear samples. **Figures 10A, B** show the evolution process of the regression coefficients of master model A in the independent SS-FPME method and the sequential SS-FPME method, respectively. This illustration is helpful to better understand the batch adjustment of the regression coefficients. Compared with the regression coefficient of master model A, the regression coefficient of the updated batch B model was basically the same as that of batch A, whereas the regression coefficients of the updated batch C and D models varied greatly, thus improving the prediction performance of the model. The difference of regression coefficients was unique for each batch of samples. It was difficult, however, to extract information related to chemical composition to analyze the causes of these spectral changes.

Although we used the same microfiber optic spectrometer to collect the NIR spectra and followed the same standard procedures to measure the LC reference for each batch of samples, the performance of detecting the LC of the batch B, C, and D samples using master model A was poor, with lower Rp values and higher RMSEP values. The varieties in the NIR spectra of the samples occurred for several potential reasons, including changes in the detector light source, the acquisition environment temperature, the operation of spectral collection and reference value determination, and the process and equipment of the sample pretreatment. In this study, **Table 5** shows that the updated model using the SS-FPME method based on the batch A samples could improve the performance of predicting the LC of the batch B, C, and D samples. Compared with the independent SS-FPME method used to update the master model, the sequential SS-FPME method could enhance the model features from previous supervised learning and obtain better prediction perfosssrmance. Therefore, the updated model based on supervision and learning of a new batch sample using the sequential SS-FPME method could improve the robustness and migration ability of the model to detect the LC of snow pears and provided technical support for the development of a portable detection device.

**Table 5:** Robustness of different updated model based on NIR spectroscopy for detecting LC in snow pear.

Batch of samples	Method	Rp	RMSEP
B	Model A directly predict batch B	0.824	0.641
	Ind-model AB predict batch B	0.837	0.614
C	Model A directly predict batch C	0.602	1.703
	Ind-model AC predict batch C	0.940	0.433
	Seq-model ABC predict batch C	0.952	0.383
D	Model A directly predict batch D	0.413	0.917
	Ind-model AD predict batch D	0.806	0.332
	Seq-model ABCD predict batch D	0.831	0.309

## 4 Conclusion

We examined the robustness of the calibration model used to predict the LC of different batches of snow pears based on NIR spectroscopy. The results showed that the performance of the calibration model updated using the SS-FPME method with a small number of samples from a new batch of snow pears was improved. The NIR spectra at nine different measurement positions of snow pear samples purchased at four different periods were collected by a microfiber optic spectrometer. Then, the average NIR spectra of each sample in batch A were processed by 1-Der (11), 2-Der (11), SNV, and MSC pretreatment methods. Next, we selected 19, 76, 80, 24, and 20 effective wavelengths and compared them to build a model to predict the LC of snow pears using SPA, SiPLS, GA, BOSS, and CARS variable selection methods, respectively. As a result, the SNV-GA-PLSR model (master model A) had higher Rc and Rp values of 0.846 and 0.854, lower RMSECV and RMSEP values of 0.637 and 0.624, and the difference between the Rc and Rp and the RMSECV and RMSEP were also smaller. Thus, this model was used as the prediction model for detecting the LC in the other three batches of snow pear samples. Although we used the same microfiber optic spectrometer to collect the NIR spectra and followed the same standard procedures to measure the LC reference for each batch of samples, the performance of detecting the LC of the batch B, C, and D samples by the master model A was poor, with lower Rp values and higher RMSEP values. We used and compared the independent SS-FPME method and the sequential SS-FPME method to update master model A for predicting the LC of snow pears.

For the batch B samples, the predictive ability of the updated model (Ind-model AB) was improved: the Rp value increased from 0.823 to 0.837, and the RMSEP value decreased from 0.641 to 0.614. For the batch C samples, the performance of the Seq-model ABC was improved greatly: the Rp value increased from 0.602 to 0.952, and the RMSEP value decreased from 1.703 to 0.383. For the batch D samples, the performance of the Seq-model ABCD was also improved: the Rp value increased from 0.413 to 0.831, and the RMSEP value decreased from 0.916 to 0.309. Moreover, the prediction performance of the updated model based on the sequential SS-FPME method was better than that of independent SS-FPME method, which indicated that the sequential update enhanced the model features in the learning of previous batches. Therefore, the updated model based on supervision and learning of new batch samples according to the sequential SS-FPME method improved the robustness and migration ability of model to detect the LC of snow pears and provided technical support for the development of a portable detection device.

## Data availability statement

The original contributions presented in the study are included in the article/**Supplementary Material**. Further inquiries can be directed to the corresponding author.

## Author contributions

Conceptualization, XW and GL; methodology, XW; software, XW; validation, XW, XF and WW; formal analysis, XW; investigation, XW; resources, XW; data curation, XW; writing—original draft preparation, XW; writing—review and editing, XW; visualization, XF; supervision, GL; project administration, GL; funding acquisition, XW and WW. All authors contributed to the article and approved the submitted version.
